# Current Hospital Topics

**Published:** 1906-06-23

**Authors:** 


### HOSPITAL ADMINISTRATION.

### CONSTRUCTION AND ECONOMICS.

### CURRENT HOSPITAL TOPICS.

#### Country Branches for City Hospitals.

The practice of establishing convalescent homes, or country branches, for the reception of patients suffering from open wounds, in connection with the city hospitals, is extending to the United States. When it is remembered that, on a reasonable estimate, the reduction in the number of days hospital residence required after operations to secure the return of a condition of health enabling work to be resumed represents the addition of one million days every year to the wage earning power of the working classes of London alone, the value of the country branch becomes inestimable. The fact that, after a given number of days, a severe operation case is found to halt when the patient is kept in a city hospital bed, and that when the patient is moved out to the country he rapidly and completely recovers as a rule, is now demonstrably proved. Its recognition is causing the average stay of each patient under hospital care to be reduced from thirty-five days to nineteen days or less, with the further advantage that the cases sent to the country are returned to their families in a condition of health which enables them to resume their occupations without risk. Formerly many patients were sent out from the hospitals before they were physically able to resume their ordinary work.

#### Charity Accounts and Business Firms.

Sir James Blyth has repeated in the *Times* his proposal of three years ago that all business firms should open a charity account in their ledgers; that they should specially earmark a definite proportion of their profits for the credit of that account; and that much of the total each year should be distributed amongst the voluntary hospitals. He suggests one per cent. on the profits, which he estimates for the United Kingdom, on the income-tax returns, to exceed £600,000,000 sterling a year. If Sir James Blyth's suggestion were generally adopted it would yield a revenue of six millions per annum, of which he thinks one half might be contributed in annual subscriptions to the hospitals. Sir James maintains that in well-organised commercial houses there is already a charity account, from which a regular payment is made to the funds of the hospitals out of the profits on the business. These charity accounts are regarded as being upon the same footing as a pension fund, for the staff employés of such firms may and do largely participate in the benefits of the hospitals. For these reasons Sir James appeals to all business men, as a matter of charitable business, to increasingly systematise the giving to hospitals, and so to escape from the impassioned appeals which they at present receive. No doubt systematic giving is an excellent habit for business men, and, indeed, for all intelligent members of the community. Sir James has, however, been unduly alarmed, apparently, by the "imploring and almost despairing tone of the varied and daily appeals for help to hospitals" which he has received. We are all for business methods in the conduct of our hospitals. If, however, the hospitals are to deserve and receive the support of business men on some such plan as Sir James Blyth has suggested, it is essential that the "imploring and despairing tone" of a few, and we rejoice to know, a decreasing number of hospital appeals in these days, which is sometimes so exaggerated as to perilously approach, if it does not even amount in fact, to untruth, must be permanently abandoned.

#### The Municipal or Rate-Supported Hospital Delusion.

Speaking at the laying of the foundation-stone of the School of Advanced Medical Studies at University College Hospital, Sir Donald Currie regretted that there was so much difficulty in obtaining support for the many hospitals in this country, and that the burden of maintenance should be thrown upon a few benevolent people. These did much to establish hospitals and to support them; but he felt that the day would come, and might be close at hand, when the ratepayers or taxpayers of this country would require to do their part to uphold these institutions. Last week we gave extracts from the report of the superintendent of a rate-supported hospital at Cincinnati, which showed that under such management a branch devoted to tuberculosis was overcrowded with patients, and that the atmosphere of its wards was not only highly detrimental to the afflicted, but a menace to those who minister to the patients, for they are compelled to breathe the disease-laden atmosphere. We further showed that in the infectious branch of the same hospital the superintendent pointed out the great injustice of compelling decent, law-abiding citizens to commingle in the same wards with criminals and some of the lowest of the low. Our English experience of rate-supported hospitals has not been altogether a happy one, and it is a fact, as the history of the world proves, that if the whole of the English hospitals were to be supported by the rates, there is no question that they might soon lose their reputation for being on the whole the most efficiently conducted hospitals in the world. They would further be liable to sink as to their administration to a dead and monotonous level of relative inefficiency, and the lot of the patients, in such circumstances, might often prove pitiable indeed. Fortunately, as we showed in this column in our issue of May 26 last, Sir Donald Currie is unduly pessimistic in speaking of the volume of ɔoluntary support extended to the London hospitals as a whole.

It is true, no doubt, that University College Hospital and one or two others, though they are a small minority, are financially in a most unsatisfactory condition. The financial position of University College Hospital, however, must not be taken as indicative of the average position of the best-managed and more prosperous voluntary hospitals of London. It does but represent a condition of affairs which has been brought about by causes that are fully recognised and known to those who have followed the history of the metropolitan hospital system in recent years. No doubt the financial weakness of Charing Cross, St. Mary's, and University College Hospitals must be speedily tackled energetically, but the remedy for the existing difficulties is not far to seek, and will no doubt be applied in due course. One swallow does not make a summer, and a few specially needy, financially weak hospitals, out of nearly a hundred, must not be taken as indicating that the voluntary system of hospital support is gradually failing. We can assure Sir Donald Currie that there never was a notion more widely divorced from the actual facts than that which rests upon the assertion that financially the voluntary system of support as applied to British hospitals is so inadequate as to render a resort to rate support either necessary in the present or even probable in the remote future.

